# Associations of Genes for Killer Cell Immunoglobulin-like Receptors and Their Human Leukocyte Antigen-A/B/C Ligands with Abdominal Aortic Aneurysm

**DOI:** 10.3390/cells10123357

**Published:** 2021-11-30

**Authors:** Joanna Dubis, Wanda Niepiekło-Miniewska, Natalia Jędruchniewicz, Maciej Sobczyński, Wojciech Witkiewicz, Norbert Zapotoczny, Piotr Kuśnierczyk

**Affiliations:** 1 Research and Development Centre, Regional Specialist Hospital, 51-124 Wroclaw, Poland; natalia.jedruchniewicz@wssk.wroc.pl; 2 Laboratory of Immunogenetics and Tissue Immunology, Hirszfeld Institute of Immunology and Experimental Therapy, Polish Academy of Sciences, 53-114 Wrocław, Poland; wanda.niepieklo-miniewska@hirszfeld.pl; 3 Laboratory of Molecular Neurobiology, Nencki Institute of Experimental Biology, Polish Academy of Sciences, 02-093 Warsaw, Poland; m.sobczynski@nencki.edu.pl; 4 Department of Vascular Surgery, Regional Specialist Hospital in Wroclaw, 51-124 Wrocław, Poland; witkiewicz@wssk.wroc.pl (W.W.); nzapotoczny@gmail.com (N.Z.)

**Keywords:** abdominal aortic aneurism, genetics, KIR, HLA, immune cells, aneurysm diameter, CAD, BMI

## Abstract

Abdominal aortic aneurysm (AAA) is an immune-mediated disease with a genetic component. The multifactorial pathophysiology is not clear and there is still no pharmacotherapy to slow the growth of aneurysms. The signal integration of cell-surface KIRs (killer cell immunoglobulin-like receptors) with HLA (ligands, human leukocyte class I antigen molecules) modulates the activity of natural killer immune cells. The genetic diversity of the KIR/HLA system is associated with the risk of immune disorders. This study was a multivariate analysis of the association between genetic variants of KIRs, HLA ligands, clinical data and AAA formation. Genotyping was performed by single polymerase chain reaction with sequence-specific primers using commercial assays. Patients with HLA-A-Bw4 have a larger aneurysm by an average of 4 mm (*p* = 0.008). We observed a relationship between aneurysm diameter and BMI in patients with AAA and co-existing CAD; its shape was determined by the presence of HLA-A-Bw4. There was also a nearly 10% difference in KIR3DL1 allele frequency between the study and control groups. High expression of the cell surface receptor KIR3DL1 may protect, to some extent, against AAA. The presence of HLA-A-Bw4 may affect the rate of aneurysm growth and represents a potential regional pathogenetic risk of autoimmune injury to the aneurysmal aorta.

## 1. Introduction

An abdominal aortic aneurysm (AAA) is defined as a local permanent dilatation of the aorta with a diameter of >30 mm that occurs mostly in the infra-renal region [1]. The main risk factors are male sex and older age of patients [1,2]. The prevalence of AAA in men aged 65–74 years is approximately 2.7%; it increases with age and it is an important public health problem [2,3,4]. As Eastern European populations age, the AAA prevalence is expected to increase significantly [1,3]. AAA usually remains asymptomatic until it ruptures; this occurrence represents a mortality risk for about 80% of patients [5]. In the absence of pharmacotherapy, to prevent progressive growth or rupture of the AAA, advanced surgical treatment is the only option [4]. Therefore, screening for aneurysms and early detection of local lesions remain fundamental to the management of this disease [1,2,3,4]. AAA is associated with coronary artery disease (CAD), the body mass index (BMI) and smoking, among other factors; despite intensive research, the pathogenesis of the disease is still unclear [4,5,6].

The AAA pathophysiology is multifactorial, with several genetic factors acting at different developmental stages of the disease [7,8,9]. The AAA is accompanied by endothelial dysfunction, loss of integrity and thinning of the vessel wall and increased inflammation [4,7]. It is characterised by extensive tissue remodelling with protease-mediated degradation of the extracellular matrix and depletion of vascular smooth muscle cells [4,9]. The disease development is accompanied by activation of the immune system: chronic inflammatory cells infiltrate the aneurysm wall [4,8,10]. Many different immune mononuclear cells in the aneurysmal tissue, including natural killer (NK) cells, natural killer T cells (NKTs) and B and T lymphocytes produce cytokines and enzymes promoting inflammation and extracellular matrix degradation and, thereby, contribute to aortic dilatation, progression and uncontrolled rupture of an AAA [4,7,10]. It is likely that aortic damage and autoantigen exposure further contribute to the tissue destruction [11]. The inflammatory infiltrate in the AAA wall contains immunoglobulins, cytokines and proteases, all factors that implicate the activation of host innate and adaptive immune responses [11,12,13,14,15,16]. Considerable research has provided strong evidence that autoimmune processes are involved in the development of the AAA and contribute to the progressive degradation of the aorta [11,14,15,16]. A genome-wide association study using gene expression microarrays and the immunohistochemical analysis of aneurysmal tissue revealed significant involvement of NK cell-mediated cytotoxicity in the pathogenesis of human AAA [12,17]. NK cells are a heterogeneous population of innate lymphoid cells, primarily localised in tissues (5–15% are found in the bloodstream). Recent studies have highlighted that, independent of innate cytotoxicity (associated with the direct release of cytolytic factors), NK cells are involved in shaping adaptive immune responses and the generation of memory responses by the secretion of a number of cytokines and chemokines [18,19,20].

The cytotoxic activity of NK cells is regulated by the integration of signals derived from activating and inhibitory receptors expressed on their surface [21,22,23]. Prominent among these are the killer cell immunoglobulin-like receptors (KIRs), which are encoded by a diverse family of KIR genes clustered on chromosome 19q13, one of the most variable regions of the human genome [22,23]. The region encompassing KIR genes shows extensive variability in terms of both gene content and sequence polymorphism. The KIRs are characterised by the presence of two (2D) or three (3D) extracellular immunoglobulin-like domains and either a long (L) or short (S) intracellular domain, which is distinguished in the nomenclature [21]. Specific KIRs recognise subsets of the major histocompatibility complex (MHC) class I molecules known as the human leukocyte antigen (HLA) molecules, namely HLA-A, HLA-B and HLA-C [23,24]. Upon interaction with HLA ligands on target cells, KIR receptors with short or long intercellular domain produce activating or inhibitory signals, respectively, which regulate the cytotoxic function of these immune cells [23]. The interactions between KIR activating/inhibitory receptors and HLA class I gene products determine the activation threshold for cytotoxic cells [21,24].

HLA class I molecules are present on the surface of all nucleated cells in the body and play a critical role in the recognition of infected or transformed cells and the acquisition of competence by NK cells [19,21,24,25]. The signal integration of cell-surface KIRs with HLA ligands allow NK cells to discriminate ‘altered self’ from ‘normal self’. NK cell cytotoxic activity is tightly controlled by inhibitory and activating receptors to prevent an inappropriate response against host cells. This allows potentially self-destructive NK cell activity to be kept in check. The loss or downregulation of HLA class I molecules in damaged, transformed or infected cells renders host cells ‘foreign’ and thus susceptible to killing by NK cells [21,25]. The acquisition of functional competence by NK cells takes place in an education process (licensing) involving both inhibitory and activating receptors and in the presence of their HLA ligands [22,25,26].

HLA are encoded on chromosome 6p21.3 and are inherited independently from KIRs, which are encoded by 19q13.4. The combined inheritance of highly polymorphic *KIR* and *HLA* genes (*KIR/HLA*) within the human population causes a wide variety of immune responses to pathological conditions [21,23,24]. Genetic diversity of the KIR/HLA system is associated strongly with the risk of immune system abnormalities and has implications for the prevalence, progression and outcome of many conditions with inflammatory causes and autoimmune diseases [22,25,27]. 

AAA is a life-threatening immune disease with chronic inflammation, the pathogenesis of which is still unknown. Thus, the aim of the present study was to analyse the association between genetic variants of KIRs, their HLA ligands and the formation of an AAA.

## 2. Materials and Methods

Nomenclature for the *KIR* genotypes was applied in accordance with the Allele Frequencies Website (http://www.allelefrequencies.net/, accessed on 28 October 2021). The planned study was performed in accordance with the principles of the Declaration of Helsinki and was approved by the local Bioethics Committee (KB/nr15/2010), and informed consent was obtained from all participants.

### 2.1. Study Population

We enrolled 187 patients who underwent elective surgical repair of AAA with aneurysm ≥40 mm (subjects with aneurysms diameter <40 mm were not included). All patients were admitted to the Research and Development Centre of the Regional Specialist Hospital in Wroclaw. The size of aorta in the study groups was assessed by ultrasonography. Before surgery, the AAA was diagnosed in detail by computed tomography, which can detect dilatation of the abdominal aorta. All the diagnoses were confirmed by an intraoperative assessment. The clinical and demographic characteristics of our cohorts are shown in Table 1.

The control group included 229 individuals who took part in a trial of ultrasound screening for AAA. Normal diameter was defined as maximum infrarenal aortic diameter ≤30 mm. A control group were matched for age, gender, smoking habits and comorbidities. Patients and controls in whom a history of cancer, immunological and autoimmunological disorders was ascertained in the medical interview were excluded from the study. 

### 2.2. KIR and HLA genotyping

Genomic DNA of examined individuals was isolated from peripheral EDTA-anticoagulated blood using QIAamp^®^DNA Blood Mini Kit (Qiagen, Hilden, Germany), according to the manufacturer’s instructions. Genotyping of *KIR* and *HLA-A,B,C-*encoded KIR ligand genes was performed by single polymerase chain reaction with sequence-specific primers by using commercial tests (Olerup SSP KIR Genotyping and Olerup SSP KIR HLA Ligand, Olerup SSP AB, Stockholm, Sweden.), in line with manufacturer’s instructions. Genomic DNA from both patients and controls was typed for the presence of the KIR genes: *2DL1*, *2DL2*, *2DL3*, *2DL4*, *2DL5A*, *2DL5B*, *3DL1*, *3DL2*, *3DL3*, *2DS1*, *2DS2*, *2DS3*, *2DS4full*: (2DS4*0010101-00103), *2DS4del*: (2DS4*003, *004, *006, *007, *008, *009), *2DS5*, *3DS1*, *2DP1*, *3DP1*. 

Two main groups of *KIR3DL1* alleles were analysed: the alleles highly expressed on the cell surface (*KIR3DL1High*): 3DL1*001-002, *008, *009, *015, *020, and alleles encoding receptors with low or negligible expression on the cell surface, (KIR3DL1Low): 3DL1*004, *005, *019; the alleles *022-035, *038, *040-044, *051-054, *057, *059-068, *073 were included by the test in the first group, and alleles *021, *036, *037, *039, *056, *072 in the second group [Olerup SSP^®^ KIR Genotyping Product Description], although their levels of cell surface expression is not known yet. Alleles *005 and *007 were placed in the first group by our test, although their expression is rather low [28,29].

The following *HLA* genes were designed for the KIR ligands: *HLA-C* Asparagine 80 (*HLA-C1*), *HLA-C* Lysine 80 (*HLA-C2*), *HLA-B-Bw4-*Threonine 80 (*HLA-B-Bw4-80T*), HLA-B-Bw4-Isoleucine 80 (*HLA-B-Bw4-80I*) and *HLA-A-Bw4*. 

The *KIR* genotyping used in this study has been validated three times per year by the International KIR Exchange Program organized by the Immunogenetics Center of the University of California in Los Angeles. 

### 2.3. Statistical Analysis

To investigate the relationship of clinical, demographical and genetic variables with the probability of onset of AAA and the aorta’s diameter, generalized linear models with binomial and Gaussian errors were used, respectively. The global test for difference between two sets of dependent proportions, i.e., $x_{1}={\left(p_{11},\;\ldots ,\;p_{1k}\right)}^{T}$ and $x_{2}={\left(p_{21},\;\ldots ,\;p_{2k}\right)}^{T}$ was, $T=\frac{\|x_{1}-x_{2}{\|}_{2}}{SE\left(\|x_{1}-x_{2}{\|}_{2}\right)}$, where distribution of statistic was estimated with Monte Carlo simulation. Akaike’s information criterion was used as a measure of the model fit. The bootstrap approach was employed to estimate the model’s coefficients and 95% confidence intervals. The chi-square test was used to test hypotheses at count data in tables. Divergence from homogeneity of two distributions tested with chi-square test was measured with standardized Pearson’s residuals. The *odds ratio* (OR) was computed as a measure of effect size. The $S_{n}$ statistic was computed as the measure of variability: $S_{n}=med\left\{med\left|x_{i}-x_{j}\right|;i,j=1,\ldots ,n\right\}$, when median was used as a measure of central tendency. $S_{n}$ is typical difference between two randomly selected observations. The KIR haplotype frequencies (HFs) were estimated with the *maximum likelihood* function. Group A and B haplotypes were considered, leading to the following genotype classification: genotype A/A: *KIR2DL1*, *KIR2DL3*, *KIR2DL4*, *KIR3DL1* (any allele), *KIR3DL2*, *KIR2DS4* (any allele); genotype A/B: all group A genes plus at least one additional *KIR* gene such as *KIR2DS1*, *KIR3DS1*, *KIR2DS2*, *KIR2DS3*, or *KIR2DS5*; genotype B/B: group A genes (except *KIR3DL1*, present in almost all individuals) missing and group B genes *(KIR2DS1*, *KIR3DS1*, *KIR2DS2*, *KIR2DS3*, and/or *KIR2DS5*) present. Association between two variables in tables 2 × 2 was measured with Yule’s *Q* statistic $Q=\frac{n_{11}n_{22}-n_{12}n_{21}}{n_{11}n_{22}+n_{12}n_{21}}\in \left[-1,1\right]$, where Q = 0 means independence of two variable to each other and homogeneity of two compared distributions. Homogeneity of two or more odds ratios was tested with Breslow–Day procedure. Results were regarded as statistically significant at *p* < 0.05. 

## 3. Results

In this study, allelic polymorphism of *KIR* genes and their ligands (*HLA-A*, *B*, *C)* were analysed to find links between *KIR* locus and formation of AAA in study groups. The absence/presence polymorphism of inhibitory and activating KIRs was noted. The incidence of common haplotypes A and B in the studied groups was also determined. Moreover, variations in the interaction of immunoglobulin-like receptors and their ligands *HLA-A*, *B*, *C* were analysed in patients with AAA and compared with the control group. 

### 3.1. Frequency of KIRs and HLA Ligands in AAA Patients and Controls

First, genotyping of *KIR* genes was carried out in all recruited subjects. The number of individuals carrying each *KIR* gene and the individual gene frequencies for tested KIRs in study and control groups is demonstrated in Table 2. A lower frequency of the *KIR3DL1High* allele (63.1% vs. 72.9%) and a higher frequency of the *KIR3DL1Low* (32.2% vs. 22.7%) allele were observed in the AAA group when compared to control. We observed a nearly 10% difference in the frequency of *KIR3DL1* allele groups among the study cohorts. 

For both of these *KIR* genes, *KIR3DL1High* and *KIR3DL1Low,* presented in Table 2, confidence intervals CI95% suggest that differences in frequencies of these genes in AAA patients and controls are statistically significant. In case of *KIR3DL1High* presence the risk of disease was 1.6 times lower (Table 2: OR = 0.63, CI95 = 0.42; 0.96, *p* = 0.0324) comparing to a person without this gene. We estimated additionally the risk of AAA with (OR = 0.61) and without the presence of HLA-A-Bw4 ligand (OR = 0.65). This result suggest that the risk of AAA is independent from the HLA-A-Bw4 presence. In case of *KIR3DL1Low* (Table 2: OR = 1.77, CI95 = 1.15; 2.73, *p* = 0.00954)*,* the presence of which is negatively correlated with the presence of the *KIR3DL1High* (Yule’s measure of association Q=−0.997 in Controls and Q=−1 in AAA), the risk of AAA is OR = 1.9 with presence of HLA-A-Bw4 and OR = 1.7 without it. The difference between these two odds ratios is not significant (*p* = 0.802) and the results again suggest that the risk of AAA associated to the presence of KIR3DL1 genes is not dependent on the presence of HLA-A-Bw4 ligand. In Supplementary materials we present additionally Table S1 with frequencies of four possible pairs KIR3DL1/HLA-A-Bw4 among the AAA and control group. Based on chi-square test, we estimated residuals which measure divergence from the homogeneity of the distributions in two groups. In the AAA group we can see, that in case of *KIR3DL1High* residual for the subgroup of patients without both KIR3DL1 and HLA- is $r_{-/-}=1.44$ and residual for subgroup without KIR3DL1 but with HLA-A-Bw4 present $r_{-/+}=1.35$, which means that, in the AAA group, there were more people without *KIR3DL1High* then expected, and it confirms protective effect of this gene. What is more, this protective effect is invariant of presence of HLA-A-Bw4 ligand. Similarly, in case of *KIR3DL1Low* $r_{+/-}=1.75$ and $r_{+/+}=1.68$ for AAA, which means that in the AAA group there were more people with *KIR3DL1Low* then expected and it confirms again higher risk of AAA associated with presence of *KIR3DL1Low,* independently from the presence of HLA-A-Bw4 ligand. 

Both *KIR3DL1* gene variants seem to be significantly associated with the risk of AAA, but one must take into account that these results were obtained after 14 *KIR* genes had been tested, so caution must be preserved. That is why we performed additionally global omnibus test for all tested *KIR* genes presented in Table 2. The statistical analysis shows that there is no convincing evidence that the frequency of individual *KIR* genes is significantly different in patients with AAA compared with those without aneurysms (T = 4.641, *p* = 0.1513). No differences were found, either, after taking into account the presence of HLA ligands and clinical data in the study groups ($\chi_{df=14}^{2}=1.168$ *p* = 0.6181). The multivariate statistical analysis performed did not take into account some of the genes (*KIR2DL4, KIR3DL2, KIR3DL3*, *KIR2DS, KIR2DP1*, and *KIR3DP1*). The *KIR2DL4* and *KIR3DP1* genes were not included because they were found in all patients with AAA and in almost all of the control group with the exception of one person. The *KIR3DL2*, *KIR3DL3* and *KIR2DP1* genes were detected in all subjects. 

Subsequently, the samples tested for *KIR* genes were genotyped to determine the frequencies of the HLA epitopes which are responsible for the binding of individual KIRs (HLA-C1, C2, B-Bw4-80T, B-Bw4-80I, A-Bw4). The distribution of the HLA ligands in analysed groups is presented in Table 3. There were no significant differences in the frequencies of the analysed *HLA* genes between the compared groups (T = 2.063, *p* = 0.8109). 

The frequencies of *KIR* (except for alleles *KIR3DL1High* and *KIR3DL1Low*, not tested elsewhere in Poles) and *HLA* genes in our cohorts were similar to the frequencies reported earlier in the Polish population [30,31,32].

### 3.2. KIR A/B Genotypes and Haplotypes 

All subjects were identified as KIR AA, AB and BB genotypes depending on the presence of activating and inhibitory genes. Analysis did not show significant differences between the frequencies of these genotypes in the patient cohort compared with the control group ($\chi_{df=2}^{2}=1.48$; *p* = 0.476). The KIR AB genotypes in both the study group and control group (59.9% and 54.1%, respectively) outnumbered the AA (27.3% and 30.1%) and BB (12.8% and 15.7%) genotypes (Table 3). Based on the observed genotypes, the frequency of haplotypes in the study groups was estimated. In the group of patients with AAA, the haplotype with the *KIR3DL1Low* allele occurs more frequently than in the control group (16.25% vs. 11.25%), whereas the haplotype with the *KIR3DL1High* allele occurs at a lower frequency (25.42% vs. 20.83%, Table 4). 

### 3.3. Aneurysm Diameter, KIR Genes, HLA Ligands and Clinical Parameters 

**Figure 1:**
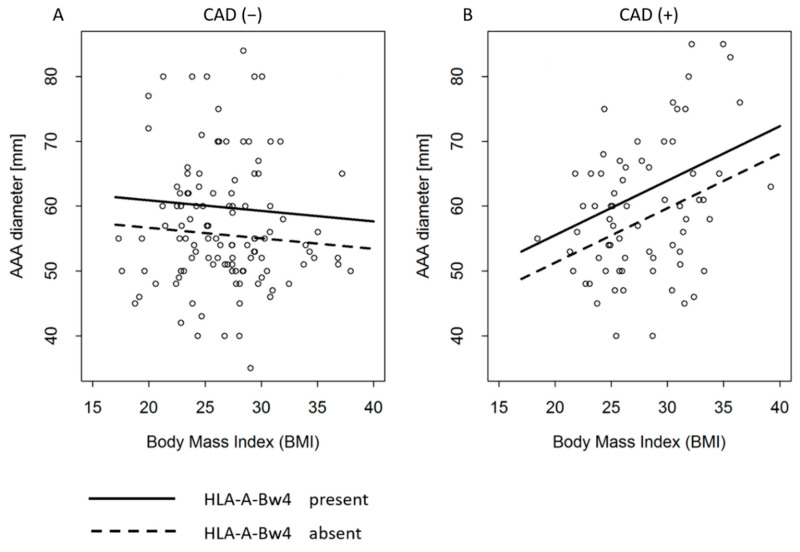
Relation between BMI and AAA diameter, according to CAD and HLA-A-Bw4 presentation (*p* = 0.0005). (**A**) AAA patients with no CAD, (**B**) AAA patients with CAD.

Table 5 presents the results of the regression analysis of AAA diameter. We can see that there are three variables which influence the AAA diameter: presence of HLA-A-Bw4, presence of CAD, BMI and interaction of BMI and CAD marked here as BMI × CAD. We start analysis of the Table 5 from the last factor, i.e., interaction between BMI and CAD. This significant interaction is a consequence of the fact, that AAA diameter depends on BMI but only in patients with CAD (*p* = 0.004); this can be seen on the B plot on Figure 1. The regression coefficient for the interaction BMI × CAD is equal to β = 1.003 and is positive which means that among patients with CAD, higher BMI is associated with higher AAA diameter, and lower BMI with lower AAA diameter. The coefficient β = 1.003 means that with every 1 kg/m^2^ BMI, the expected AAA diameter is β = 1.003 mm larger. There is no such relationship in group of patients without CAD (*p* = 0.392, A plot Figure 1). 

**Figure 2:**
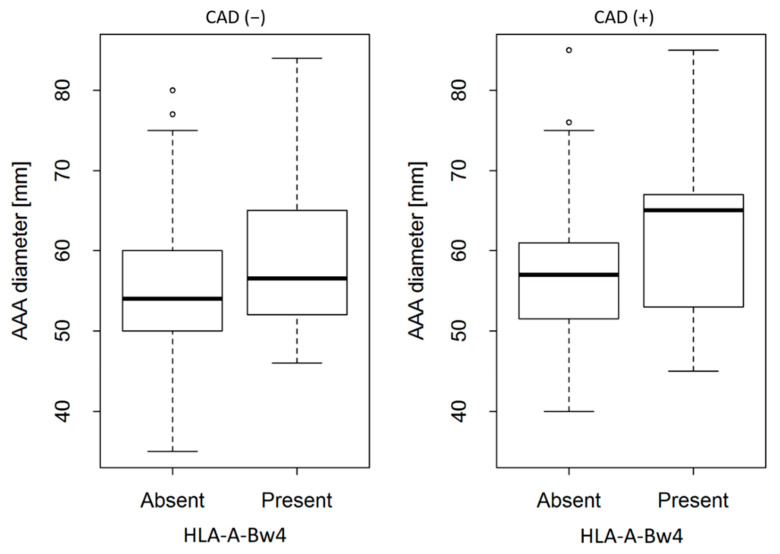
Differences between AAA diameter subdivided according to the presence of HLA-A-Bw4 and CAD (*p* = 0.008). For CAD (−) and CAD (+), see legend to Figure 1.

The second factor strongly related to AAA diameter is the presence of HLA-A-Bw4. A group of patients possessing HLA-A-Bw4 had a mean of AAA diameter β = 4.250 millimetres larger than group of patients with the same BMI and the same CAD status but without HLA-A-Bw4 (*p* = 0.008). This factor influences the diameter independently from BMI and CAD, i.e., the presence of HLA-A-Bw4 always increases the AAA diameter. It can be seen in the Figure 1 where the regression solids line are expected AAA diameter for patients with HLA-A-Bw4 and it is higher than the dotted lines, which represent expected AAA diameter for patients without HLA-A-Bw4. It can be also seen in the Figure 2. Another important observation shown in the Table 5 is the negative coefficient for CAD equal to β = −25.463. It means that AAA diameter in patients with CAD tends to be smaller than in the population of patients without CAD. However, the significant interaction between BMI and CAD is positive (β = 1.003) what means that CAD effect is reduced in patients with high BMI. In other words, the higher the BMI, the weaker the “protective” CAD effect. Neither KIRs nor their ligands had relationship with AAA diameter (*p* = 0.7058).

## 4. Discussion

This paper presents, for the first time, the results of analysis of the association of genes encoding KIRs and their HLA ligands with AAA. An analysis was performed on the study and control group in relation to the presence and allelic polymorphism of functional *KIR* genes and their major *HLA-A*, *B*, *C* ligands, taking into account the clinical and demographic data of the individuals included in the study. 

We have demonstrated that aneurysm diameter significantly depends on the presence of the *KIR* ligand group *HLA-A-Bw4* allele. A detailed analysis including genetic determinants and clinical data of patients showed significant correlations between aneurysm diameter, HLA-A-Bw4, CAD and BMI. These findings suggest that the presence of CAD and BMI may modulate the relationship between aneurysm size and HLA-A-Bw4 during disease progression. 

On the other hand, multivariate statistical analysis of our results indicates that the incidence of AAA does not depend on the frequency of polymorphic *KIR* genes and their major ligands *HLA-A*, *B*, *C* except for the *KIR3DL1* gene’s high- and low-expressed variants. 

An AAA is now thought to result from a synergism between systemic immune imbalance and local autoimmunity [11,14]. The data published to date provide evidence that AAA is specific antigen-driven T-cell autoimmune disease [15]. Several autoantigens have been suggested to be involved in the development of AAA, among them collagen, elastin and fibrinogen [11,16]. Antiphospholipid antibodies typical of autoimmune diseases were also identified in AAA patients [11]. Furthermore, the presence of AAA nonself (microbial) antigens implies that molecular mimicry may be responsible for the T-cell response in patients [11,16]. Many studies suggest that innate NK immunity is significantly involved in the development of autoimmune responses and chronic inflammation in AAA tissues [11,12,13]. 

Aneurysm tissue undergoes progressive change during development under the influence of genetic and environmental factors and is, therefore, a special environment for NK cells. Local environmental changes are related to both blood flow biomechanics and aortic degradation [33]. In the lumen of the aneurysm, thrombus and calcifications occur, resulting in a change in the biomechanics of blood flow from laminal to pulsatile [33,34]. Different heterogeneous subgroups of NK cells may appear locally at given stages of AAA development. Thus, in the early stage of the disease the NK immune response may be different from that in the advanced stage. 

The presented result of the association of the HLA-A-Bw4 molecule with aneurysm size seems to be related to local inflammation and autoimmunity in the affected aorta. The HLA system is currently being studied in terms of autoimmunity in AAA patients, but the research is mainly focused on understanding the expression of HLA class II in this group of patients [35,36]. However, the data so far are inconclusive. There are documented links of the allele HLA-DQA1*0102 with the occurrence of AAA [35]. In addition, HLA-DRB1*01 and HLA-DRB1*16 have been suggested to be involved [36]. However, little is known about the role of HLA class I molecules in the formation and development of AAA. The HLA-A*02 and HLA-A*61 alleles have been shown to be the probably genetic risk factors for AAA [37]. An association between the presence of HLA-B*68 and the diameter of AAA has also been reported, but to date no convincing evidence has been provided to support these results [38]. 

HLA class I molecules containing the Bw4 motif on the alpha helix (allotypes: HLA-A-Bw4+ and HLA-B-Bw4+) are ligands for the inhibitory receptor KIR3DL1 [23,39]. The inhibitory receptor KIR3DL1 plays key roles in both mediating cytolysis and NK licensing (education) [21,22]. It is one of the most interesting surface receptors of the KIR family, since it is encoded by a gene with a high prevalence in the population: only approx. 5% of the population does not have any of its alleles, and those negative for KIR3DL1 possess activating KIR3DS1, which behaves as its allele. 

The interaction of HLA-Bw4+ allotypes with KIR3DL1 occurs via the Bw4 epitope, with an amino acid at position 80 of the α1 HLA helix determining this interaction [39]. HLA-B allotypes with Bw4 have either an isoleucine or threonine in position 80 (HLA-B-80I or HLA-B-80T), so they can bind to KIR3DL1 with different strength; as shown by protein structure studies, this dimorphism (80I/80T) determines an array of binding strengths, leading from strong to weak inhibition and licencing of NK cells. All *HLA-A-Bw4* alleles have isoleucine residue (80I) in this position [39]. In addition, the binding strength also depends also on the KIR3DL1 allotype, as well as on the peptide bound by Bw4-positive class I HLA molecule, making the interpretation of both experimental and clinical data even more complicated [39]. Recently, the inhibition of NK cells by HLA-A-Bw4/KIR3DL1 interaction has been also shown to be diverse and hierarchical: only HLA-A*32 binds KIRDL1 strongly and educate NK cells, whereas HLA-A*23 and HLA-A*24 bind weakly, and HLA-A*25 does not bind at all [39,40,41]. Studies, in vitro, performed on NK cells from healthy donors and transfected cell lines have shown that HLA-A-Bw4 (HLA-A*23, HLA-A*24, A*25 and HLA-A*32) molecules are differentially expressed on the cell surface and exhibit different capacities to educate and inhibit NK. The HLA-A*24 and HLA-A*32 allotypes show the strongest ability to inhibit NK using KIR3DL1, whereas HLA-A*25:01 lacks a capacity to inhibit KIR3DL1+ NK cells [39,40]. 

The strength of HLA-A-Bw4 binding to the KIR3DL1 receptor (affinity) is clinically relevant. For example, interaction of KIR3DL1 receptor and HLA-A*24 is associated with risk of acute myelogenous leukemia relapse after allogeneic hematopoietic cell transplantation [40]. HLA-A-Bw4 epitope was found to be associated with diseases, in the course of which inflammation is increased. The epitope is associated with psoriasis, where KIR3DS1/HLA-A-Bw4 genotype was positively associated with disease susceptibility and KIR3DL1/HLA-B-Bw4-80I was negatively associated [42]. KIR3DL1/HLA-Bw4 axis influences also patients’ response to anticancer drugs based on antibodies with specificity directed against cancer cells. The presence of the HLA-A-Bw4 isoform or HLA-B-Bw4-80T isoform is associated with the improved anti-tumour response to monoclonal antibody-based immunotherapy, as compared with HLA-B-Bw4-80I [43]. This finding is surprising, because HLA-A-Bw4 is 80I, not 80T [39]. However, in the HLA-A-Bw4/KIR3DL1 interaction, other amino acid residues within and outside the Bw4 motif sequence also play a role; as Saunders et al. 2021 write, “differential recognition across HLA-Bw4 allotypes is therefore likely a product of polymorphic residues in both the a1 and a2 domains, differences in peptide repertoire and conformation, as well as KIR3DL1 allotype” [39]. The inhibitory KIR3DL1 is one of the most polymorphic alleles of all the KIR loci and is divided into three allotype groups: KIR3DL1Null (*004, *019), which show no expression at the cell surface, KIR3DL1Low (*005, *007) showing low expression, and KIR3DL1High (*001, *002, *008, *009, *015, *020) exhibiting high expression at the cell surface [28,29,44,45]. However, our test did not differentiate exactly between these groups, particularly between KIR3DL1Null and KIR3DL1Low, dividing KIR3DL1 into two groups only. Therefore, we designated KIR3DL1 alleles as KIR3DL1High or KIR3DL1Low (encompassing both KIR3DL1Low and KIR3DL1Null alleles) (see Materials and Methods). The comparative analysis presented in this paper showed opposite associations of the two groups of *KIR3DL1* gene variants (*KIR3DL1High* and *KIR3DL1Low*) with AAA: a protection associated with *KIR3DL1High* and risk associated with *KIR3DL1Low*. The obtained result had no statistical significance after correction for multiple comparisons, but odds ratios nevertheless suggest the participation of KIR3DL1 low versus high expressors in the complex pathogenesis of AAA. Expression profiling studies by Lenk and colleagues showed that *KIR3DL1* gene expression is detectable in AAA tissue but not in normal aorta [17]. It suggested that cells with a KIR3DL1+ phenotype may be involved in local immune processes in the aneurysm.

Considering that AAA patients have a lower frequency of high-expression normal receptor (*KIR3DL1High* alleles) and a higher frequency of receptor not or only weakly expressed on the cell surface (*KIR3DL1Low*), it would be expected that the NK inhibitory function is impaired in this group of patients. It is possible to speculate that the integrated inhibitory signal, due to the KIR3DL1Low/HLA-Bw4+ interaction, is weakened and, thus, NK cells are activated. 

In the present study, an inverse association between aneurysm diameter and CAD incidence was demonstrated in patients with AAA. The result confirms the data presented by Nakayama et al. regarding the inverse relationship between AAA progression and CAD [46]. Our study showed a positive association between aneurysm diameter and BMI in patients CAD (+). It is difficult to explain this relationship due to the lack of research in this area. One can only speculate that anti-CAD drugs, which perhaps indirectly reduce AAA progression, are less effective in obese individuals. Our observation confirms multifactorial background of AAA development and requires detailed explanation and requires a detailed explanation at the cellular level. 

Interestingly, *KIR3DL1Low* alleles are distributed, in most populations, with quite remarkable frequencies [www.allefrequencies.net, accessed on 28 October 2021] which speaks in favour of some role also for the non-expressed or low-expressed alleles. The null *KIR3DL1* allele is expressed exclusively intracellularly, which prevents the inhibition of NK by Bw4-expressing target cells, but does not preclude education [44]. Several studies suggest that the pathogenesis of inflammatory and autoimmune diseases is associated with variability between patients in NK education [25]. In the presence of activating factors, such as antibodies on target cells or a pro-inflammatory microenvironment, uneducated NK cells can be rapidly stimulated to kill target cells [22,25]. 

Interactions of KIR3DL1 with HLA-A-Bw4 allotypes are poorly understood compared to HLA-B-Bw4. Polymorphic KIR receptors binding *HLA-A*, *B*, *C* molecules, and conserved NKG2A/CD94 receptor binding HLA-E on target cells, are responsible for NK education and their response to the presence of transformed or infected cells. HLA-E molecule becomes a ligand for NKG2A/CD94 upon binding a peptide cleaved from a leader sequence of these HLA class I molecules which possess methionine in position −21. This residue facilitates the folding and HLA-E expression on the cell surface [25,47]. All HLA-A and HLA-C molecules contain this favourable leader sequences for HLA-E (i.e., methionine −21), which enable efficient education of NK cells with the participation of a conserved NKG2A/CD94 receptor. However, methionine −21 is present only in a minority of HLA-B allotypes. Threonine −21, a residue present in most HLA-B allotypes, does not allow the peptide from the leader sequence to bind efficiently to HLA-E, and promotes NK education and maturation by KIRs. It is a relatively recent acquisition in primate evolution, present in the MHC-B of humans, chimpanzees and gorillas but not the lower monkeys, except in some rare cases of probably independent mutations [47]. These *HLA-A-Bw4*-positive alleles, which interact with KIR3DL1 (i.e., all except A*25 [39,40,41]) form haplotypes with threonine −21 *HLA-B* alleles 3.5 times more frequently than with methionine −21 HLA-B, at least in Poles (Table 6) [30]. This may explain why we observed AAA association with KIR3DL1 alleles, whose products, by interaction with HLA-A-Bw4-linked HLA-B-21Thr+ alleles, contributed to NK cell education via KIRs, making these cells functionally mature and using KIRs, including KIR3DL1. The other haplotypes, also containing HLA-B alleles with −21 threonine but not HLA-A-Bw4 alleles, may educate NK cells via KIRs too. This may explain why the risk of AAA, in our study, was associated with KIR3DL1 recognizing the Bw4 epitope on both HLA-A and HLA-B but was not associated with HLA-A-Bw4. Of course, the association with HLA-A-Bw4, if existing, might have been overlooked because our typing test did not differentiate between KIR3DL1-recognized (i.e., A*24, A*23 and A*32) and -non-recognized (A*25) HLA-A-Bw4 alleles. 

Our finding that a risk of AAA is associated with KIR3DL1Low alleles but not with the KIR3DL1 ligand, HLA-Bw4 (on HLA-A or HLA-B molecule), while the size of diameter of aneurism is associated with HLA-A-Bw4 but not with its receptor, KIR3DL1 (neither high nor low expressor allotype) needs explanation. KIR3DL-Low alleles are quite frequent in Caucasian populations [28,29,48] (www.allelefrequencies.net, accessed on 28 October 2021), and were even more frequent in our AAA patients, and at least some of them must have contained only null alleles. Therefore, in these individuals the presence or absence of a ligand presumably did not play a role. This could explain a lack of HLA-A-Bw4 (and also HLA-B-Bw4) association with disease risk. On the other hand, once AAA has started, during ongoing inflammation the expression of HLA molecules, including HLA-A-Bw4 ones, may be increased by IFN-γ and other cytokines, hence the visible influence of HLA-A-Bw4 presence. 

The reason why AAA diameter was associated with HLA-A-Bw4 but not with KIR3DL1 (neither high nor low expressors) seems more difficult to explain. We may assume, however, that *HLA-A-Bw4* alleles, where A*23 and A*24 have the same peptide-binding motif different from other class I HLA alleles [49], contribute to later stages of AAA, expressed in its enlarged diameter, by presenting antigenic peptide to CD8+ T cells rather than by stimulating NK cells. This might explain why only HLA-A-Bw4, but not HLA-B-Bw4+ molecules seem to play a role, here. 

There are certain limitations to this work. First, the study group was comprised of people with a relatively large AAA (>40 mm) who had qualified for surgery. Second, there is a comparatively small number of women in the study and the control group. The inclusion of people with a small AAAs would allow us to verify our results in a group of people at an early stage of the disease. Increasing the number of women in the study groups could provide new, interesting results. Moreover, including more people in the study and control group would increase the power of statistical tests.

## 5. Conclusions

**Figure 3:**
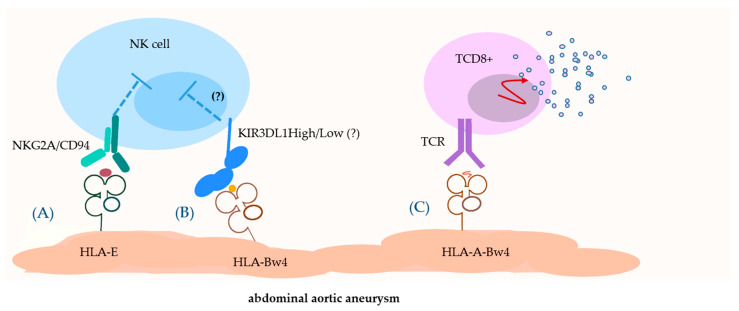
The scheme illustrating hypothetical role of HLA-A-Bw4 molecule and KIR3DL1 receptor in immunopathogenesis of AAA. (**A**) The non-classical HLA-E may display a peptide derived from the leader sequence of HLA-A molecules. HLA-E/HLA-A peptide complex interacts with NKG2A/CD94 on immune cells (NK, TCD8+) and drives their education. (**B**) The HLA-Bw4 (both HLA-A-Bw4 and HLA-B-Bw4) molecule interacts with KIR3DL1 and inhibits the activity of immune cells. (**C**) The HLA-A-Bw4 molecule presenting the aneurysmogenic peptide interacts with the TCR receptor, which leads to the activation of cytotoxic lymphocytes.

Our preliminary results show for the first time that:

1. The risk of AAA may be associated with KIR3DL1 because majority of HLA-B allotypes contain the threonine residue in position −21 of their leader sequence, which prevents it from being presented by HLA-E molecule and NK education by NKG2D/CD94 receptor, but favours education via KIRs, including KIR3DL1. HLA-A-Bw4 alleles occur most frequently in linkage disequilibrium with *HLA-B* alleles containing −21 threonine, with the same result as above (Figure 3A). Therefore, we could not detect any role of the Bw4 epitope, on HLA-A or on HLA-B, in the initiation of AAA, for three reasons: (a) the signal for education of NK cells is focused on position −21 of HLA-B, not on Bw4. (b) AAA might be initiated by KIR3DL1Low rather than KIR3DL1High because the too-weak inhibition by this KIR favours the activation of NK cells (Figure 3B). (c) Our test system did not differentiate between HLA-A-Bw4 strong binders of KIR3DL1 (A*32), weak binders (A*23, A*24) and no binders (A*25). Therefore, a contribution of strong versus weak binders might have been masked, to some extent, by weak and no binders.
2. On the other hand, AAA diameter was associated with *HLA-A-Bw4* alleles, and not with *KIR3DL1* or with *HLA-B-Bw4*. It is conceivable that at this stage the contribution of HLA-A-Bw4 allotypes presenting aneurysmogenic peptide to CD8+ T cells (Figure 3C) is more important than the KIR3DL1-Bw4 interaction, which in turn is important for regulation of NK cell activity. This interpretation should be confirmed on larger cohorts of patients and controls, and, optimally, by high-resolution typing of both *HLA-A-Bw4* and *KIR3DL1* alleles, as well as by functional studies.

## Figures and Tables

**Table 1:** Demographic and clinical characteristics AAA patients (*n* = 187) and control subjects (*n* = 229).

	Median ± S_n_ (Range) or Number of AAA Patients	Median ± S_n_ (Range) or Number of Controls
age [years]	72 ± 7 (43–90)	67 ± 5 (58–84)
sex [male]	167 (89.3%)	220 (96.1%)
smoking	122 (65.2%)	89 (38.9%)
BMI [kg/m^2^]	26.9 ± 3.8 (17.3–39.2)	27± 3.1 (19.6–38.7)
CAD	70 (37.4%)	25 (10.9%)
hypertension	149 (79.7%)	34 (14.8%)
kidney disease	34 (18.2%)	38 (16.6%)
diabetes	40 (21.4%)	101 (44%)
AAA diameter [mm]	56± 8 (40–105)	-
aortic diameter [mm]	-	19.2 ± 2.2 (14–28)

S_n,_ measure of variability in case when median is used as measure of central tendency. AAA, abdominal aortic aneurysm; BMI, body mass index; CAD, coronary artery disease.

**Table 2:** KIR genes frequency in AAA patients and controls.

KIR	AAA, *n* = 187	Controls, *n* = 229	OR (95%CI)
Inhibitory			
*2DL1*	185 (98.9%)	222 (96.5%)	2.92 (0.60–14.2)
*2DL2*	110 (58.8%)	118 (51.5%)	1.34 (0.91–1.98)
*2DL3*	170 (90.9%)	209 (91.3%)	0.96 (0.49–1.88)
*2DL5A*	61 (32.6%)	88 (38.4%)	0.78 (0.52–1.16)
*2DL5B*	55 (29.4%)	64 (27.9%)	1.07 (0.70–1.65)
*3DL1High ^a^*	**118 (63.1%)**	**167 (72.9%)**	0.63 (0.42–0.96)
*3DL1Low ^b^*	**64 (34.2%)**	**52 (22.7%)**	1.77 (1.15–2.73)
Activating			
*2DS1*	74 (39.6%)	103 (45.0%)	0.80 (0.54–1.19)
*2DS2*	108 (57,8%)	116 (50.7%)	1.33 (0.90–1.96)
*2DS3*	64 (34.2%)	79 (34.5%)	0.99 (0.66–1.48)
*2DS4full*	68 (36.4%)	73 (31.9%)	1.22 (0.81–1.84)
*2DS4del*	158 (84.5%)	193 (84.3%)	1.02 (0.60–1.73)
*2DS5*	44 (23.5%)	57 (24.9%)	0.93 (0.59–1.46)
*3DS1*	68 (36.4%)	90 (39.3%)	0.88 (0.59–1.32)

T = 4.641; *p* = 0.1531. No significant differences after clinical characteristics adjustment ($\chi_{df=13}^{2}=1.089$; *p* = 0.6041). ^a^ Cell-membrane highly expressed *KIR3DL1* alleles: *0010101-002, *0060101-0060102, *007-010, *012, *013 ^b^. Alleles lowly or not expressed on the cell membrane: *00401-00403, *019, *021, *036, *037, *039, *056, *072 ^a, b^. The low-resolution test does not allow to differentiate between non-expressed homozygotes and heterozygotes of these two groups of alleles. These two groups of *KIR3DL1* alleles do not add up to 100% because some individuals may have *KIR3DS1* instead. Abbreviations: AAA, abdominal aortic, aneurysm; CI, confidence interval; KIR, killer immunoglobulin-like receptor; *KIR2DS4fl*, *KIR2DS4* full length gene; *KIR2DS4del*, 22-base pair deletion variant of the *KIR2DS4* gene; OR (95% CI), odds ratio with 95% confidence interval (CI).

**Table 3:** Ligands and KIR-genotypes frequency in cases and controls.

	AAA Patients	Controls	OR (95%CI)
HLA Ligand *			
*C1*	151 (80.7%)	176 (76.9%)	1.26 (0.78–2.03)
*C2*	121 (64.7%)	156 (68.1%)	0.86 (0.57–1.29)
*B-Bw4-80I*	50 (26.7%)	68 (29.7%)	0.86 (0.56–1.33)
*B-Bw4-80T*	56 (29.9%)	72 (31.4%)	0.93 (0.61–1.42)
*A-Bw4*	60 (32.1%)	69 (30.1%)	1.10 (0.72–1.66)
Genotypes **			
A/A	51 (27.3%)	69 (30.1%)	0.87 (0.57–1.33)
A/B	112 (59.9%)	124 (54.1%)	1.26 (0.86–1.87)
B/B	24 (12.8%)	36 (15.7%)	0.79 (0.45–1.38)

* T = 2.063; *p* = 0.8109, ** $\chi_{df=2}^{2}=1.48$; *p* = 0.476.

**Table 4:** Estimated KIR haplotypes frequencies in cases and controls.

Cases (%)	Controls (%)	Centromeric Region						Telomeric Region							
		*2DS2*	*2DL3*	*2DL2*	*2DL5B*	*2DS3*	*2DL1*	*3DL1High*	*3DL1Low*	*3DS1*	*2DL5A*	*2DS5*	*2DS1B*	*2DS4A*	*2DS4B*
20.83	25.42	0	1	0	0	0	1	1	0	0	0	0	0	0	1
16.25	11.25	0	1	0	0	0	1	0	1	0	0	0	0	0	1
5.78	6.2	0	1	0	0	0	1	0	0	1	1	1	1	0	0
2.74	5.99	1	0	1	0	0	0	1	0	0	0	0	0	0	1
8.95	5.13	0	1	0	0	0	1	1	0	0	0	0	0	1	0
3.48	3.25	1	0	1	1	1	1	0	0	1	1	0	1	0	0
0.34	3.07	1	0	1	0	0	1	0	0	1	1	1	1	0	0
0.07	2.73	0	1	0	0	0	1	0	0	0	0	0	0	0	1
4.16	2.42	1	0	1	1	1	1	1	0	0	0	0	0	0	1

**Table 5:** Variables related to AAA diameter. Results of regression analysis.

Variable	β	CI95%		*p*-Value
HLA-A-Bw4 CAD	4.250	1.251	7.423	0.008
	−25.463	−44.562	−7.681	0.007
BMI	−0.162	−0.548	0.201	0.392
CAD × BMI	1.003	0.344	1.707	0.004

R^2^ = 0.0866, F_4;179_ = 5.34; *p* = 0.0005. Intercept = 59.9. CAD, coronary artery disease; BMI, body mass index; β, regression coefficient.

**Table 6:** Frequencies of HLA-A-Bw4, HLA-B haplotypes and amino acid residue −21 in HLA-B leader peptide.

HLA-A-Bw4 ^a^	HLA-B	Amino Acid −21 in HLA-B Leader Peptide ^b^	Frequency in Polish Population (%) ^c^
*23:01	*44:03	threonine	0.939
*24:02	*13:02	threonine	0.892
*24:02	*07:02	methionine	0.670
*24:02	*44:03	threonine	0.277
*24:02	*15:01	threonine	0.249
*25:01	*18:01	threonine	2.489

^a^ HLA-A-Bw4-positive alleles: Rese, M. et al.; Epitope-specificities of HLA antibodies: the effect of epitope structure on Luminex technique-dependent antibody reactivity. *Hum Immunol.* 2015, 76, 297–300 [41]. ^b^ Amino acid residue in position −21 of the HLA-B leader peptide: Horowitz, A. et al. Class I HLA haplotypes form two schools that educate NK cells in different ways. *Sci Immunol.* 2016, 1(3) [47]. ^c^ HLA-A, HLA-B haplotype frequencies: Schmidt, A.H. et al. High-resolution human leukocyte antigen allele and haplotype frequencies of the Polish population based on 20,653 stem cell donors. *Hum Immunol.* 2011, 72, 558–565 [30].

## Data Availability

Not applicable.

## Supplementary Materials

The following are available online at https://www.mdpi.com/article/10.3390/cells10123357/s1, Table S1: Frequencies of KIR3DL1 with and without HLA-A-Bw4 ligand in AAA patients and controls.
