# Supplementary material for: Associations of Genes for Killer Cell Immunoglobulin-like Receptors and Their Human Leukocyte Antigen-A/B/C Ligands with Abdominal Aortic Aneurysm
Source: Cells. 2021 Nov 30;10(12):3357. doi: 10.3390/cells10123357 (PMC8699266; doi:10.3390/cells10123357)
Supplement: Supplementary file 1 [file cells-10-03357-s001.zip › cells-1461810-supplementary.pdf]

Table S1. Frequencies of *KIR3DL1* with and without *HLA-A-Bw4* ligand in AAA patients and controls. Annotations: -/- in case of *KIR3DL1* absent and *HLA-A* absent; +/- in case of *KIR3DL1* present and *HLA-A* absent and so on.

KIR3DLHigh/HLA-A-Bw4					
	**-/-**	**+/-**	**-/+**	**+/+**	Sum
Controls	43	117	19	50	229
%	18.8	51.1	8.3	21.8	100%
AAA	46	81	23	37	187
%	24.6	43.3	12.3	19.8	100%
Standardized Pearson residuals					
Controls	-1.44	1.58	-1.35	0.51	-
AAA	**1.44**	-1.58	**1.35**	-0.51	-
$\chi^2_{df=3} = 4.78; p = 0.1888$					
K3DL1Low/HLA-A-Bw4					
	**-/-**	**+/-**	**-/+**	**+/+**	Sum
Controls	124	36	53	16	229
%	54.1	15.7	23.1	7.0	100%
AAA	85	42	38	22	187
%	45.5	22.5	20.3	11.8	100%
Standardized Pearson residuals					
Controls	1.76	-1.75	0.69	-1.68	-
AAA	-1.76	**1.75**	-0.69	**1.68**	-
$\chi^2_{df=3} = 6.99; p = 0.0722$					
